# Systematic Review of Mucosal Immunity Induced by Oral and Inactivated Poliovirus Vaccines against Virus Shedding following Oral Poliovirus Challenge

**DOI:** 10.1371/journal.ppat.1002599

**Published:** 2012-04-19

**Authors:** Thomas R. Hird, Nicholas C. Grassly

**Affiliations:** Department of Infectious Disease Epidemiology, Imperial College London, Norfolk Place, London, United Kingdom; University of California San Francisco, United States of America

## Abstract

Inactivated poliovirus vaccine (IPV) may be used in mass vaccination campaigns during the final stages of polio eradication. It is also likely to be adopted by many countries following the coordinated global cessation of vaccination with oral poliovirus vaccine (OPV) after eradication. The success of IPV in the control of poliomyelitis outbreaks will depend on the degree of nasopharyngeal and intestinal mucosal immunity induced against poliovirus infection. We performed a systematic review of studies published through May 2011 that recorded the prevalence of poliovirus shedding in stool samples or nasopharyngeal secretions collected 5–30 days after a “challenge” dose of OPV. Studies were combined in a meta-analysis of the odds of shedding among children vaccinated according to IPV, OPV, and combination schedules. We identified 31 studies of shedding in stool and four in nasopharyngeal samples that met the inclusion criteria. Individuals vaccinated with OPV were protected against infection and shedding of poliovirus in stool samples collected after challenge compared with unvaccinated individuals (summary odds ratio [OR] for shedding 0.13 (95% confidence interval [CI] 0.08–0.24)). In contrast, IPV provided no protection against shedding compared with unvaccinated individuals (summary OR 0.81 [95% CI 0.59–1.11]) or when given in addition to OPV, compared with individuals given OPV alone (summary OR 1.14 [95% CI 0.82–1.58]). There were insufficient studies of nasopharyngeal shedding to draw a conclusion. IPV does not induce sufficient intestinal mucosal immunity to reduce the prevalence of fecal poliovirus shedding after challenge, although there was some evidence that it can reduce the quantity of virus shed. The impact of IPV on poliovirus transmission in countries where fecal-oral spread is common is unknown but is likely to be limited compared with OPV.

## Introduction

The development and licensing of inactivated poliovirus vaccine (IPV) in 1955 and subsequently of the live-attenuated oral poliovirus vaccine (OPV) in 1961 had an enormous impact on poliomyelitis in the Western world and raised the possibility of global eradication [1]. In 1988 the World Health Assembly adopted a resolution to eradicate poliomyelitis, which led to a successful global programme that has reduced the number of children paralysed by poliomyelitis from approximately 350,000 each year to 1,349 in 2010. Eradication of poliomyelitis though the use of these vaccines relies on herd immunity, whereby unimmunized children are less likely to become infected because neighboring children have been vaccinated. Eradication is achieved even if all children have not been successfully immunized so long as the average number of secondary infections generated by each infected individual (the “reproduction number”) is less than 1.

Critically important to the herd immunity effect is the degree of mucosal immunity offered by vaccination against infection and shedding of poliovirus. The success to date of the Global Polio Eradication Initiative (GPEI) in eliminating wild-type poliovirus transmission from most of the world can largely be ascribed to mass vaccination campaigns with OPV. This vaccine was chosen not only because of the ease of administration, but also because of its superior ability to induce local intestinal mucosal immunity [2]. Immunization with live-attenuated vaccine mimics natural infection and results in the induction of a local secretory antibody (IgA) response that is associated with a reduction in shedding of poliovirus from the intestine [3], [4]. In contrast, intramuscular injection of IPV induces serum antibodies but does not induce secretory IgA at the mucosal surfaces [3] and has a much more limited impact on the resistance of the intestine to infection [5]. However, IPV can induce gut-homing lymphocytes and an increase in the secretion of poliovirus-specific IgA among individuals who have been previously exposed to live-attenuated or wild-type poliovirus [6], [7]. The impact of this immune boosting on resistance of the intestine to infection is unknown.

After the eradication of wild-type polioviruses, coordinated global cessation of the use of OPV is envisaged to prevent vaccine-associated paralytic poliomyelitis and the emergence of vaccine-derived polioviruses [8]. The majority of higher-income and some middle-income countries that previously used OPV and have been free of indigenous wild-type poliovirus transmission for several years have already switched to IPV in their routine immunization schedules for these same reasons. At the time of OPV cessation, many other countries are likely to want to use IPV for a period of time to protect their population against potential outbreaks of vaccine-derived or wild-type poliovirus. For this reason the GPEI has supported an aggressive programme of research towards developing an “affordable” IPV. This has included dose-reduction strategies based on the addition of adjuvants, intradermal administration, or reduced schedules; development of safer poliovirus “seed” strains to allow manufacture of IPV in lower-income countries; and engagement with vaccine manufacturers to determine market size and supply capacity [9]–[11]. There have also been calls for IPV use in areas with persistent wild-type poliovirus transmission where OPV immunogenicity and effectiveness are compromised [12]. In these settings a dose of IPV could, it is argued, boost intestinal IgA better than an additional dose of OPV.

The increasingly significant role of IPV highlights the need for a better understanding of the impact of this vaccine, alone and in combination with OPV, on nasopharyngeal and intestinal mucosal immunity. Studies will be especially important in settings with efficient fecal-oral transmission of poliovirus where herd-protection through the use of IPV has never been adequately demonstrated [13].

Mucosal immunity to poliovirus in an individual can be assessed by measuring vaccine poliovirus shedding after administration of a “challenge” dose of OPV. This is considered a reasonable surrogate for immunity to infection with wild-type polioviruses after natural exposure, although the relationship between protection of the individual and prevention of transmission in the population is not well defined.

A large number of poliovirus challenge studies of variable size, location, and design have been published over the last 50 years. Although a number of clinical trials that examine the impact of IPV on mucosal immunity in tropical settings are currently under way, review of published studies from a variety of settings will also be fundamental in providing the evidence base on which countries can make their decisions about the optimal vaccination strategy—in the final stages of eradication and after global cessation of OPV use. A number of review articles have examined some of the larger OPV challenge studies [2], [14], [15], but we are not aware of any attempt at a systematic review of the large and heterogeneous group of published studies.

Here we present a systematic review of challenge studies that examine poliovirus shedding in secretions in the nasopharynx and in stool samples collected from individuals 5–30 days after administration of OPV. We present a meta-analysis of the odds of shedding poliovirus among studies that compared two or more vaccination schedules using IPV, OPV, or a combination of these vaccines. The implications for poliovirus vaccination policy are discussed.

## Results

### Identified Studies

**Figure 1:**
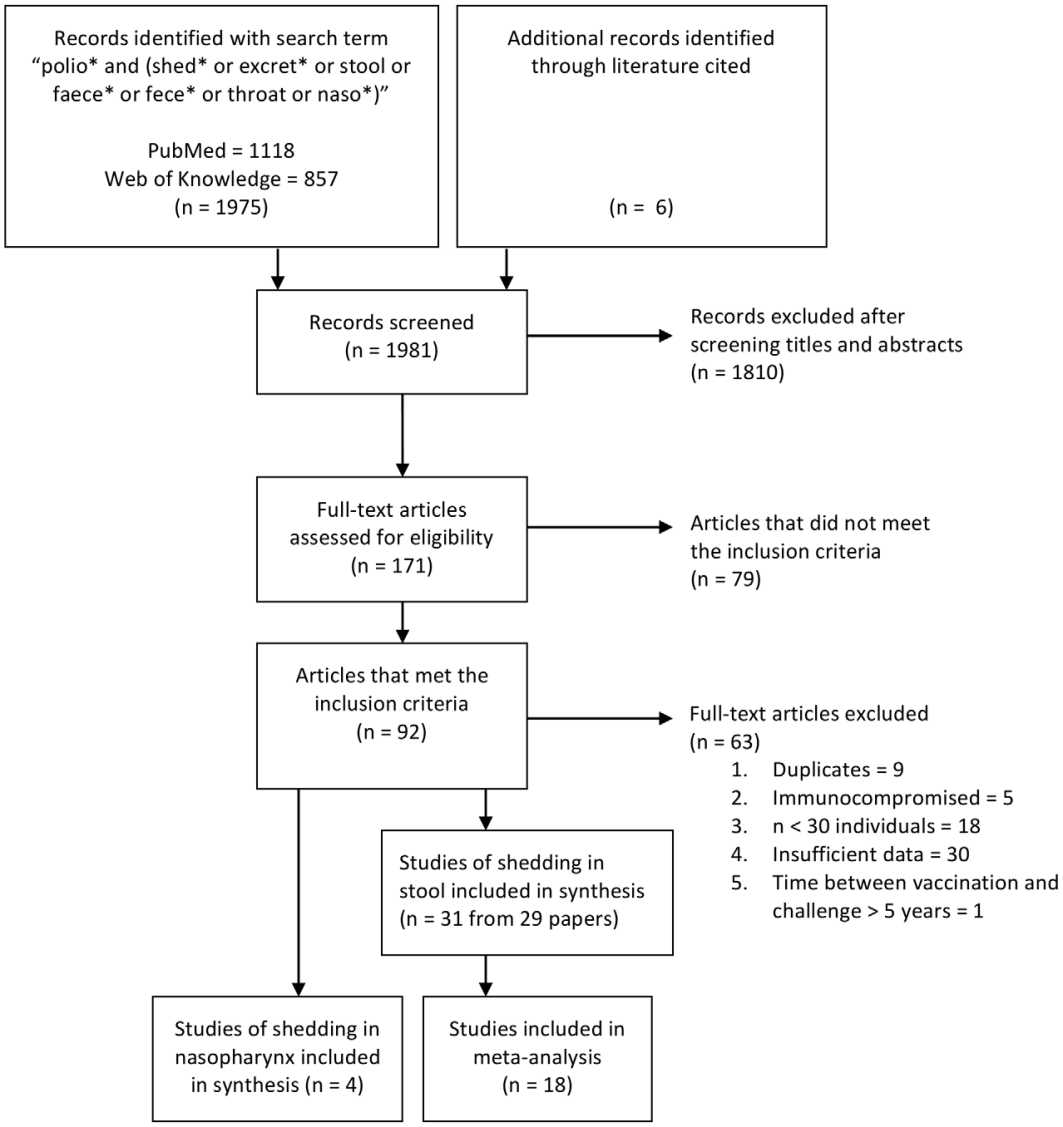
Flow diagram showing included studies according to PRISMA guidelines [**46**]. The number of published articles identified by the given search term for initial screening and the resulting studies identified and included in the systematic review and meta-analysis are shown.

A total of 1,981 published articles were identified in the PubMed and Web of Knowledge databases using the search terms described in the Methods, and a further six studies were identified from literature cited in key references (Figure 1). Screening the title and abstracts of these articles resulted in 171 potentially relevant papers, which were read in full-text to identify 31 studies of poliovirus shedding in stool and four of shedding in the nasopharynx that met the inclusion criteria for the analysis (Tables S1 and S2). One publication included studies from three different countries, and these are included in the systematic review as separate studies [16].

### Statistical Analysis

**Figure 2:**
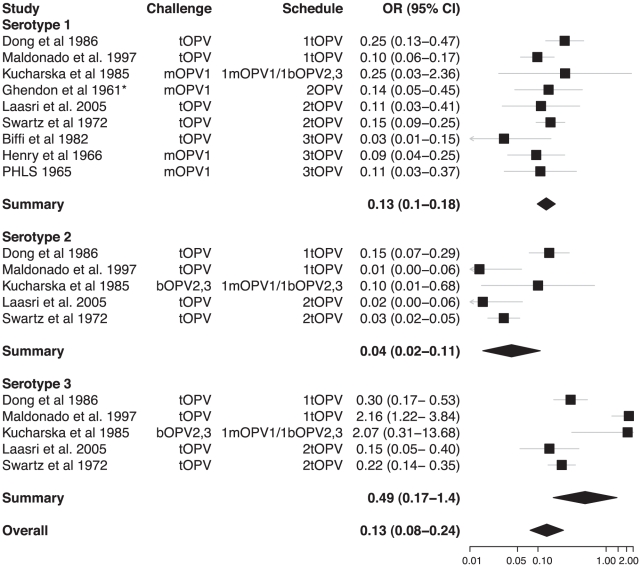
Relative odds of shedding vaccine poliovirus after challenge among individuals vaccinated with OPV compared with unvaccinated individuals. Odds ratios (ORs) and 95% confidence intervals for individual studies are indicated by the boxes and grey lines. The summary odds ratio for each serotype is given by a diamond with the 95% confidence interval (CI) indicated by its width. The χ^2^ test for heterogeneity among studies was significant for serotypes 2 and 3 (*p*-values 0.33, <0.001, and 0.001 for serotypes 1, 2, and 3, respectively) and for the overall odds ratio (*p*-value<0.001). Details of the studies included are given in Table S1. *Ghendon et al. 1961 [17] compare vaccinated and unvaccinated children who were confirmed seropositive and seronegative, respectively.

**Figure 3 ppat-1002599-g003:**
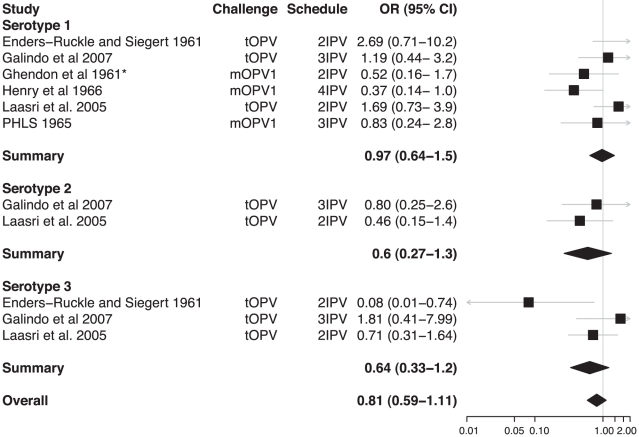
Relative odds of shedding vaccine poliovirus after challenge among individuals vaccinated with IPV compared with unvaccinated individuals. Labeling as for Figure 2. The χ^2^ test for heterogeneity among studies was not significant for any serotype (*p*-values 0.11, 0.47, and 0.07) or for the overall odds ratio (*p*-value 0.10). *Ghendon et al. 1961 [17] compare vaccinated and unvaccinated children who were confirmed seropositive and seronegative, respectively.

**Figure 4 ppat-1002599-g004:**
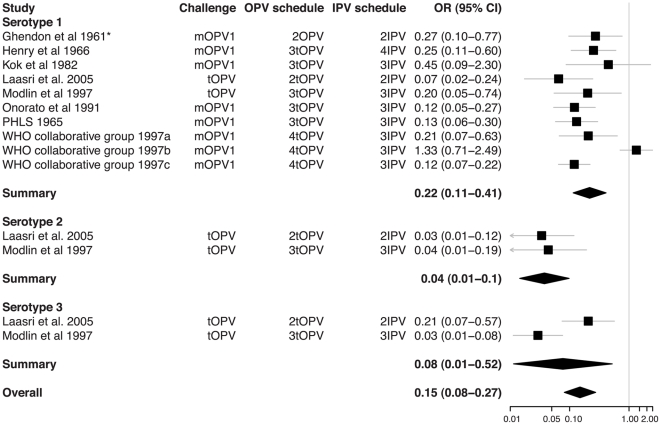
Relative odds of shedding vaccine poliovirus after challenge among individuals vaccinated with OPV compared with IPV. Labeling as for Figure 2. The χ^2^ test for heterogeneity among studies was significant for serotypes 1 and 3 (*p*-values<0.001, 0.79, and 0.01 for serotypes 1, 2, and 3, respectively) and for the overall odds ratio (*p*-value<0.001). *Ghendon et al. 1961 [17] compare vaccinated and unvaccinated children who were confirmed seropositive and seronegative, respectively.

**Figure 5:**
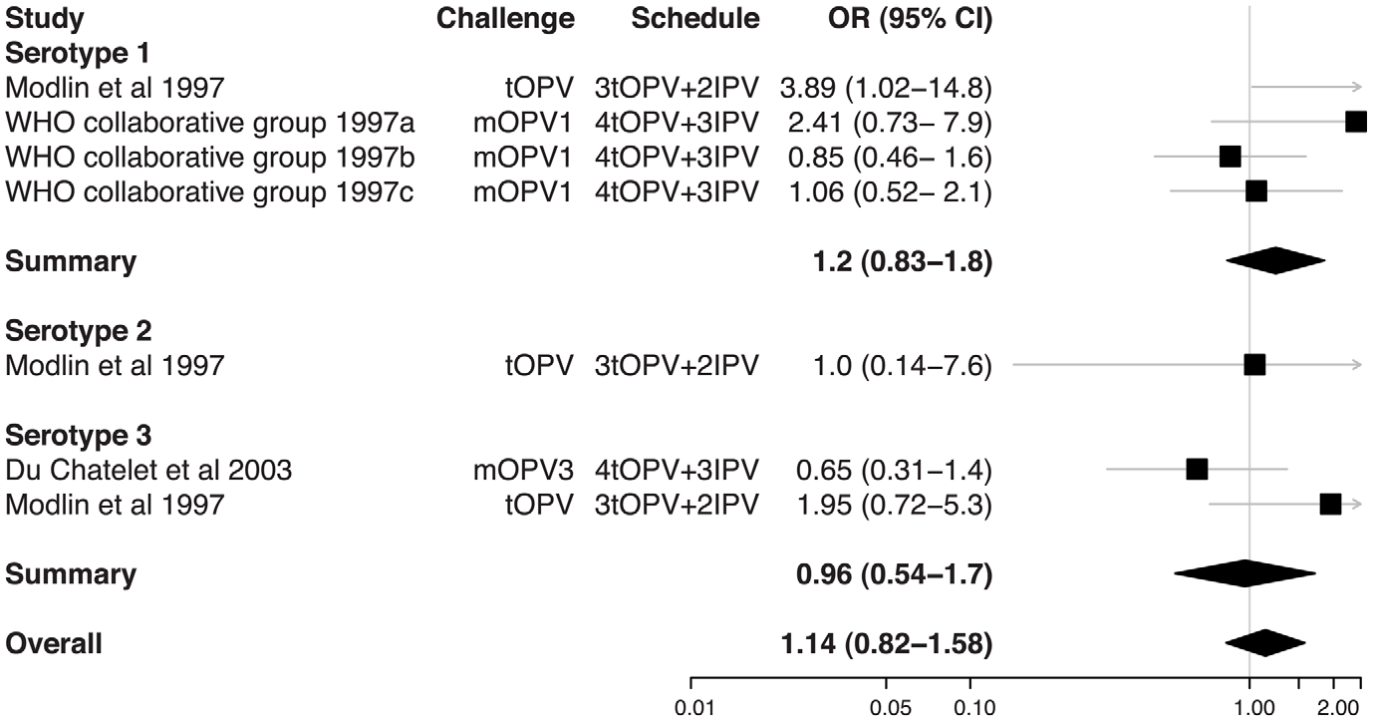
Relative odds of shedding vaccine poliovirus after challenge among individuals vaccinated with IPV in addition to OPV compared with individuals vaccinated with OPV only. Labeling as for Figure 2. The schedule indicates the number and type of OPV doses received by both groups and the number of doses of IPV that were added in the intervention group. In two studies, IPV was administered simultaneously with OPV at 6, 10, and 14 weeks (Modlin et al. 1997 [47] and du Chatelet et al. 2003 [48]), and in one study IPV was administered before and at the same time as OPV (schedule was IPV, IPV/OPV, OPV, OPV at 2, 4, 6, 15 months; WHO Collaborative Study Group on Oral and Inactivated Poliovirus Vaccines 1997 [49]). The χ^2^ test for heterogeneity among studies for serotypes 1 and 3 was not significant for each serotype (*p*-values 0.13 and 0.08) or for the serotypes combined (*p*-value 0.14).

From the 31 studies of poliovirus shedding in stool, there were 22 studies that compared shedding after challenge with the same OPV among individuals with different vaccination histories (Table S1). Classification of these vaccination histories into unvaccinated, OPV-only, IPV-only, and combined schedules permitted comparison of OPV vaccinated with unvaccinated children (Figure 2), IPV vaccinated with unvaccinated children (Figure 3), OPV with IPV vaccinated children (Figure 4), and OPV vaccinated with combined OPV/IPV vaccinated children (Figure 5; combined schedules mainly involved simultaneous administration of IPV and OPV, see figure legend for details). Summary odds ratios (ORs) for these comparisons were calculated independently for each poliovirus serotype based on fixed (*n* = 7) or random (*n* = 4) effect models according to the significance of the χ^2^ test for heterogeneity. Only one study compared serotype 2 poliovirus shedding in OPV-only and OPV/IPV vaccinated individuals, and so a summary OR was not calculated (Figure 5). There was no evidence for an association between individual study ORs and study size. In total, results from 18 studies were included in the meta-analyses that compared different vaccination histories.

Only four studies that met the inclusion criteria for the systematic review examined poliovirus shedding in the nasopharynx after administration of OPV (Table S2). Two of these studies compared IPV vaccinated with OPV vaccinated children and one compared IPV vaccinated with unvaccinated children. Very few samples were positive for poliovirus in these studies, and there was insufficient power to compare the prevalence of poliovirus in the nasopharynx of children with different vaccination histories.

## Discussion

Systematic review and meta-analysis of published studies confirms the large protective effect of prior immunization with OPV on shedding of poliovirus in the intestine following administration of a challenge dose of OPV. The odds of vaccine poliovirus shedding was significantly reduced among children immunized solely with OPV compared with unvaccinated children (overall OR 0.13 [95% CI 0.08–0.24]). In contrast, IPV had no significant impact on the prevalence of challenge poliovirus shedding in stool samples, either on its own or when added to an OPV schedule (overall ORs 0.81 [0.59–1.11] and 1.14 [0.82–1.58], respectively). The superior impact of OPV on intestinal mucosal immunity is confirmed by the meta-analysis of studies that directly compared schedules that exclusively used OPV or IPV (overall OR for OPV compared with IPV immunized children was 0.15 [0.08–0.27]).

Although IPV does not significantly reduce the prevalence of poliovirus shedding in stool samples collected after challenge, it may reduce the duration and quantity of virus shed compared with unvaccinated children. Five studies that quantified poliovirus shedding found a 63%–91% (or an absolute 0.43–1.0 log_10_) reduction in the mean quantity of poliovirus shed in stool samples collected from IPV vaccinated compared with unvaccinated children [5], [17]–[20] (Table S1). Three of these studies also examined the duration of shedding and two found a shorter period of shedding in IPV vaccinated children [17], [19]. Using data from one of these studies [17], it has been noted that the combined reduction in both the quantity and duration of vaccine poliovirus shedding would reduce the total amount of poliovirus shed during the course of an infection by approximately 95% [15]. Because IPV is unable to induce a secretory IgA response in the intestine of naïve individuals, it has been suggested that secondary exposure to OPV shed by vaccinated children or to wild-type poliovirus in the environment may have primed the mucosal immune response of children in some of these earlier studies. The effect of IPV could therefore be at least partially explained by boosting of secretory IgA among mucosally primed individuals [7]. However, the low prevalence of non-challenge poliovirus serotypes in stool samples collected during these studies suggests that mucosal priming was limited, and in the more recent study the possibility of secondary exposure to poliovirus was deliberately excluded [18]. The impact of IPV in these studies is perhaps more likely to relate to local immunity induced by IPV through transudation of serum IgG rather than induction of a local secretory IgA response [7].

There were insufficient studies that examined the impact of IPV or OPV on poliovirus shedding in the nasopharynx after administration of OPV to draw any conclusions. Three studies of wild-type poliovirus shedding in the nasopharynx after natural exposure during epidemics in the United States in 1956–1960 found a lower prevalence of shedding among children who had a history of vaccination with IPV [21]–[24]. This reduction in shedding was not apparent when stool samples were examined. However, interpretation of these studies is limited by their small size and the potential for confounding by age and socioeconomic status between IPV immunization status and the degree of exposure to wild-type poliovirus.

The relationship between reduced poliovirus shedding among vaccinated children observed in challenge studies and the impact of vaccination on wild-type or vaccine-derived poliovirus circulation is unknown and likely to vary significantly according to the characteristics of the population. Challenge with a high titer of attenuated vaccine (Sabin) poliovirus, which is homologous to the immunizing strain in the case of OPV vaccinees, is different than natural exposure to wild-type poliovirus, which has an estimated median infectious dose for humans of about 10 median tissue culture infectious doses (TCID_50_) compared with about 10^3^ for Sabin polioviruses [25]. Furthermore, the relationship between the quantity of virus shed and the probability of onwards transmission is unknown and likely to depend on the importance of different routes of transmission and dissemination in the environment.

The impact of IPV on poliovirus circulation is expected to be more limited compared with OPV in areas with poor sanitation and efficient fecal-oral transmission because of the absence of any significant effect of this vaccine on the prevalence of poliovirus shedding in stool. However, there are no studies with adequate control populations that investigate the impact of IPV on wild-type poliovirus transmission in such areas [13]. Indeed, IPV has rarely been used in lower-income countries except as part of private practice. The recent switch to routine immunization with IPV in a pilot project in Yogyakarta in Indonesia and in a number of middle-income countries in South America may provide some information about the ability of IPV to prevent circulation of vaccine-derived polioviruses in areas with poor sanitation, given the continued use of OPV in neighboring areas or during national immunization days, respectively [26], [27].

In northern European countries (France, Netherlands, Sweden, Finland, Iceland), IPV schedules have resulted in the eradication of wild-type polioviruses and protected against large outbreaks of paralytic disease for several decades [28]. The impact of IPV in these countries has been attributed to an effect of IPV on shedding in the nasopharynx in settings where oral-oral transmission is likely to predominate. Where importations of wild-type polioviruses to these countries have been documented, they have resulted in outbreaks ranging from a single case to over 100 cases of poliomyelitis [29]–[32]. These outbreaks have usually been restricted to unvaccinated communities, indicating the reduction in poliovirus transmission that results from vaccination with IPV. To date, no outbreaks have been reported from countries that have recently switched to exclusive use of IPV. However, there is some evidence from Israel that IPV-using communities are more at risk compared with OPV-using communities [33]. Furthermore, asymptomatic wild poliovirus shedding has been detected among IPV vaccinated children during outbreaks in these European countries, albeit at lower frequencies than in unvaccinated children [32], [34]. IPV vaccinated children may therefore play a role in the circulation of imported wild poliovirus, and for this reason these outbreaks have usually been controlled through the reintroduction of OPV to induce adequate mucosal immunity to stop transmission.

In some of the comparisons of vaccination schedules, the meta-analysis identified significant heterogeneity in the OR from different studies. Heterogeneity is likely to arise from a number of sources, including variable times for sample collection after challenge, different numbers and timing of vaccine doses prior to challenge, and variable laboratory procedures, as well as unmeasured factors such as the prevalence of enteric infections that may interfere with vaccine poliovirus shedding. Indeed, the prevalence of challenge poliovirus shedding was highly variable among studies, even for those that examined very similar vaccination schedules (Table S1). There were insufficient studies to permit a formal meta-regression model that included these variables. However, we did examine some of them by stratifying the meta-analysis and present the results together with the number of doses of vaccine received prior to challenge because of the association of this variable with the prevalence of shedding (Figures 2–5). For example, studies that compared poliovirus shedding among children who had received just a single dose of OPV with unvaccinated children typically found a limited impact on serotype 3 poliovirus, presumably because of the poor immunogenicity of a single dose of serotype 3 Sabin poliovirus, particularly in the trivalent formulation [35].

Despite over 50 years of vaccination with Salk's IPV, questions remain about the ability of this vaccine to prevent poliovirus circulation in remaining polio-endemic countries. In addition, basic immunology research is required to better understand the mucosal immune response to both IPV and OPV, and in particular the adaptive cellular and innate components [36], [37]. Recent evidence from India for waning intestinal immunity to poliovirus within a year of vaccination with OPV [38] and identification of wild-type polioviruses in stool samples from OPV immunized children [39] has generated interest in the potential for IPV to boost intestinal immunity among these children. Studies of immune boosting following IPV or OPV are therefore currently under way to assess the possible role for IPV in combination with OPV to interrupt wild-type poliovirus transmission in endemic countries. After eradication of wild-type polioviruses and global cessation of vaccination with OPV, the role of IPV in lower-income countries has yet to be defined. Research towards an affordable IPV aims to provide the option to use this vaccine during routine immunization and could protect children from poliomyelitis in the event of an outbreak of wild-type or vaccine-derived poliovirus. It is unknown whether this vaccine would limit the spread of poliovirus, but it would potentially provide the protection needed before an outbreak response using OPV. Continued research and programmatic use of IPV will eventually provide evidence for the impact of IPV on poliovirus circulation in countries with fecal-oral transmission of infection. It is hoped that this evidence will emerge in the context of successful global eradication of poliomyelitis.

## Materials and Methods

### Identification and Review of Studies

A literature search was carried out in May 2011 using the PubMed (http://www.ncbi.nlm.nih.gov) and ISI Web of Knowledge (http://isiknowledge.com) citation databases by searching title, abstract, and keywords with the search term “polio* and (shed* or excret* or stool or faece* or fece* or throat or naso*)”. The asterisk functions as a wildcard that permits partial word matching. We did not apply any language or publication restrictions except the restrictions of the databases themselves. Additionally, the bibliographies of key studies and reviews were examined to identify further relevant studies [2], [14], [40]. Publications in languages other than English that did not provide an English summary were translated by the authors or proficient speakers.

The titles and abstracts of articles identified by the initial search were screened and those that did not describe measures of poliovirus-specific immunity or shedding of vaccine poliovirus were removed. Full-text copies of the remaining articles were read using documents sourced from the original electronic journals or the holdings of the British Library. Data on the prevalence and quantity of vaccine poliovirus shed in nasopharyngeal or stool samples were extracted from those articles that met the inclusion criteria. These were: 1) study records the prevalence of vaccine poliovirus shedding in stool or nasopharyngeal samples collected after administration of a challenge dose of live-attenuated poliovirus; 2) samples collected 5–30 days after challenge (shedding of virus up to 4 days after challenge was excluded, because it has been suggested that this can be the result of transient passage of vaccine rather than infection of the mucosal surfaces [41]). Studies were excluded if they: 1) duplicated findings reported earlier; 2) included immuno-compromised individuals; 3) included fewer than 30 individuals; 4) included insufficient information describing poliovirus serotype, vaccine schedules prior to challenge, or prevalence of shedding by individual rather than by sample; 5) challenged with OPV more than 5 years after vaccination. These criteria ensured consistent information was available for all studies, minimizing the risk of selective reporting of favorable results within a study.

Data that were extracted from studies meeting the inclusion criteria were the vaccine type and schedule prior to challenge, challenge vaccine type and dose, the nature of the sample, and the laboratory methods (cell culture–based versus direct detection using real-time PCR). The number of individuals who shed or did not shed vaccine poliovirus was recorded by serotype and time of sample collection. Where samples were collected at multiple time points, these data were recorded separately. Data on the quantity of vaccine poliovirus shed based on titration of samples or quantitative PCR were recorded where available. We also recorded the mean duration of shedding when given or estimated this from the data where possible by taking the mean of an exponential curve fit to the prevalence of shedding over time using a least-squares approach. Data were extracted independently by the two authors and compared for errors before producing a consolidated database. Where reported data were incomplete, an effort was made to contact the authors of the relevant studies.

### Statistical Analysis

We included challenge studies that compared shedding of challenge poliovirus across two or more vaccination schedules in a meta-analysis. Where stool samples were available for more than one time point, we used data from the sample taken closest to 7 days after challenge. For the purposes of the meta-analysis, schedules were grouped into four categories—unvaccinated, trivalent OPV only, IPV only, and combined schedules—and the relative odds of poliovirus shedding calculated in pairwise comparisons between these groups. There were insufficient studies of monovalent or bivalent OPV immunization schedules to warrant a separate category for these vaccines. We only compared combined schedules with OPV or IPV-only schedules where the combination schedule involved the administration of additional doses of IPV (we did not, for example, include studies that compared a schedule of six doses of trivalent OPV with a schedule of five doses of trivalent OPV and one dose of IPV, as examined in some studies, e.g., [42]). Evidence for heterogeneity among studies was assessed on the basis of the χ^2^ statistic [43]. Summary ORs and 95% confidence intervals were calculated on a log scale assuming either fixed effects or normally distributed random effects among studies according to the results of the χ^2^ test [44]. The association between the individual study ORs and study size was examined for evidence of potential publication bias. All analyses were implemented in the R programming language using the rmeta package [45].

## Supporting Information

Checklist S1 **PRISMA statement.**
(DOC)

Table S1 **Studies included in the systematic review that examined poliovirus shedding in stool samples taken after administration of OPV.** Vaccination schedules are given as the number of doses followed by the type of vaccine. tOPV = trivalent OPV, mOPV1 = serotype 1 monovalent OPV, mOPV3 = serotype 3 monovalent OPV, bOPV2,3 = bivalent OPV containing serotypes 2 and 3. - = not available. Mean duration of shedding was estimated from the fit of an exponential curve to the prevalence of shedding over time unless given directly in the paper.
(DOCX)

Table S2 **Studies included in the systematic review that examined poliovirus shedding in nasopharyngeal secretions after administration of OPV.** Vaccination schedules are given as the number of doses followed by the type of vaccine. tOPV = trivalent OPV, mOPV1 = serotype 1 monovalent OPV.
(DOCX)
