# Supplementary material for: Systematic Review of Mucosal Immunity Induced by Oral and Inactivated Poliovirus Vaccines against Virus Shedding following Oral Poliovirus Challenge
Source: PLoS Pathog. 2012 Apr 19;8(4):e1002599. doi: 10.1371/journal.ppat.1002599 (PMC3330118; doi:10.1371/journal.ppat.1002599)
Supplement: Table S1 — Studies included in the systematic review that examined poliovirus shedding in stool samples taken after administration of OPV. Vaccination schedules are given as the number of doses followed by the type of vaccine. tOPV = trivalent OPV, mOPV1 = serotype 1 monovalent OPV, mOPV3 = serotype 3 monovalent OPV, bOPV2,3 = bivalent OPV containing serotypes 2 and 3. - = not available. Mean duration of shedding was estimated from the fit of an exponential curve to the prevalence of shedding over time unless given directly in the paper. (DOCX) [file ppat.1002599.s002.docx]

**Table S1** Studies included in the systematic review that examined poliovirus shedding in stool samples taken after administration of OPV. Vaccination schedules are given as the number of doses followed by the type of vaccine. tOPV = trivalent OPV, mOPV1 = serotype 1 monovalent OPV, mOPV3 = serotype 3 monovalent OPV, bOPV2,3 = bivalent OPV containing serotypes 2 and 3. - = not available. Mean duration of shedding was estimated from the fit of an exponential curve to the prevalence of shedding over time unless given directly in the paper.

| **Study** | **location** | **schedule** | **challenge vaccine** | **minimum titer of challenge poliovirus (log_10_ TCID_50_)** | **sample** | **detection** | **serotype** | **Proportion shedding according to sampling time % (number)** | | | | | **Time of sample collection (days after challenge)** | | | | | **Mean duration of shedding (days)** | **Mean amount of virus shed on day 7 (log10 TCID_50_/g stool unless otherwise indicated)** |
| --- | --- | --- | --- | --- | --- | --- | --- | --- | --- | --- | --- | --- | --- | --- | --- | --- | --- | --- | --- |
|  |  |  |  |  |  |  |  | **Sample number**  **1** | **2** | **3** | **4** | **5** | **Sample number**  **1** | **2** | **3** | **4** | **5** |  |  |
| Asturias et al 2007 [[1](#_ENREF_1)] | Guatemala | 2IPV | tOPV | 6 | stool | PCR | 1 | 8 (3/36) | - | - | - | - | 30 | - | - | - | - | - | - |
|  |  | 2IPV | tOPV | 5 | stool | PCR | 2 | 25 (9/36) | - | - | - | - | 30 | - | - | - | - | - | - |
|  |  | 2IPV | tOPV | 5.5 | stool | PCR | 3 | 33 (12/36) | - | - | - | - | 30 | - | - | - | - | - | - |
| Bauer et al. 1968 [[2](#_ENREF_2)] | Austria | 0-3IPV^*^ | tOPV | NA | stool | culture | 1 | 28 (24/85) | 30 (24/80) | 6 (3/53) | 15 (12/78) | 2 (1/45) | 7 | 14 | 21 | 28 | 35 | 24 | - |
|  |  | 0-3IPV^*^ | tOPV | NA | stool | culture | 2 | 28 (21/73) | 26 (19/73) | 22 (9/40) | 18 (9/48) | 7 (5/71) | 7 | 14 | 21 | 28 | 35 | 28 | - |
|  |  | 0-3IPV^*^ | tOPV | NA | stool | culture | 3 | 23 (18/78) | 11 (9/81) | 7 (4/53) | 6 (4/67) | 0 (0/68) | 7 | 14 | 21 | 28 | 35 | 11 | - |
| Biffi et al 1982 [[3](#_ENREF_3)] | Italy | unvaccinated | tOPV | - | stool | culture | 1 | 66 (23/35) | 49 (17/35) | 20 (7/35) | - | - | 1-7 | 8-14 | 14-20 | - | - | 14 | - |
|  |  | unvaccinated | tOPV | - | stool | culture | 2 | 97 (34/35) | 77 (27/35) | 29 (10/35) | - | - | 1-7 | 8-14 | 14-20 | - | - | 22 | - |
|  |  | unvaccinated | tOPV | - | stool | culture | 3 | 91 (32/35) | 63 (22/35) | 57 (20/35) | - | - | 1-7 | 8-14 | 14-20 | - | - | 28 | - |
|  |  | 3tOPV | tOPV | - | stool | culture | 1 | 6 (2/35) | 0 (0/35) | 0 (0/35) | - | - | 1-7 | 8-14 | 14-20 | - | - | 2 | - |
|  |  | 3tOPV | tOPV | - | stool | culture | 2 | 0 (0/35) | 0 (0/35) | 0 (0/35) | - | - | 1-7 | 8-14 | 14-20 | - | - | - | - |
|  |  | 3tOPV | tOPV | - | stool | culture | 3 | 0 (0/35) | 0 (0/35) | 0 (0/35) | - | - | 1-7 | 8-14 | 14-20 | - | - | - | - |
| Dong et al 1986 [[4](#_ENREF_4)] | China | unvaccinated | tOPV | 6 | stool | culture | 1 | 43 (47/109) | 10 (11/107) | - | - | - | 7 | 14 | - | - | - | 7 | - |
|  |  | unvaccinated | tOPV | 5 | stool | culture | 2 | 50 (55/109) | 21 (22/107) | - | - | - | 7 | 14 | - | - | - | 10 | - |
|  |  | unvaccinated | tOPV | 5.5 | stool | culture | 3 | 73 (80/109) | 78 (83/107) | - | - | - | 7 | 14 | - | - | - | - | - |
|  |  | 1tOPV | tOPV | 6 | stool | culture | 1 | 16 (17/108) | 5 (5/105) | - | - | - | 7 | 14 | - | - | - | 6 | - |
|  |  | 1tOPV | tOPV | 5 | stool | culture | 2 | 13 (14/108) | 8 (8/105) | - | - | - | 7 | 14 | - | - | - | 13 | - |
|  |  | 1tOPV | tOPV | 5.5 | stool | culture | 3 | 45 (49/108) | 38 (40/105) | - | - | - | 7 | 14 | - | - | - | 40 | - |
| Du Chatelet et al 2003 [[5](#_ENREF_5)] | Pakistan | 4tOPV | mOPV3 | 5.8 | stool | culture | 3 | 9 (20/233) | - | - | - | - | - | - | - | - | - | - | - |
|  |  | 4tOPV/3IPV | mOPV3 | 5.8 | stool | culture | 3 | 6 (12/209) | - | - | - | - | - | - | - | - | - | - | - |
|  |  | 4tOPV/1IPV | mOPV3 | 5.8 | stool | culture | 3 | 8 (18/213) | - | - | - | - | - | - | - | - | - | - | - |
| El-Sayed et al 2008 [[6](#_ENREF_6)] | Egypt | 1mOPV1 | mOPV1 | 6.8 | stool | culture | 1 | 26 (59/228) | 22 (50/224) | 13 (28/222) | 8 (18/220) | - | 7 | 14 | 21 | 28 | - | 20 | - |
|  |  | 1tOPV | mOPV1 | 6.8 | stool | culture | 1 | 41 (78/188) | 29 (54/184) | 15 (28/183) | 13 (24/182) | - | 7 | 14 | 21 | 28 | - | 17 | - |
| Enders-Ruckle and Siegert 1961 [[7](#_ENREF_7)] | Germany | unvaccinated | tOPV | NA | stool | culture | 1 | 39 (7/18) | 28 (5/18) | 56 (10/18) | - | - | 7 | 15 | 21 | - | - | - | median 3.5 irrespective of serotype |
|  |  | unvaccinated | tOPV | NA | stool | culture | 2 | 0 (0/18) | 0 (0/18) | 6 (1/18) | - | - | 7 | 15 | 21 | - | - | - |  |
|  |  | unvaccinated | tOPV | NA | stool | culture | 3 | 89 (16/18) | 89 (16/18) | 33 (6/18) | - | - | 7 | 15 | 21 | - | - | 36 |  |
|  |  | 2IPV | tOPV | NA | stool | culture | 1 | 63 (12/19) | 53 (9/17) | 47 (9/19) | - | - | 7 | 15 | 21 | - | - | 48 | median 2.5 irrespective of serotype |
|  |  | 2IPV | tOPV | NA | stool | culture | 2 | 0 (0/19) | 0 (0/17) | 0 (0/19) | - | - | 7 | 15 | 21 | - | - | - |  |
|  |  | 2IPV | tOPV | NA | stool | culture | 3 | 58 (11/19) | 41 (7/17) | 42 (8/19) | - | - | 7 | 15 | 21 | - | - | 39 |  |
| Galindo et al 2007 [[8](#_ENREF_8)] | Cuba | 3IPV | tOPV | 6 | stool | culture | 1 | 19 (10/52) | - | - | - | - | 7 | - | - | - | - | - | 3.46 irrespective of serotype |
|  |  | 3IPV | tOPV | 5 | stool | culture | 2 | 87 (45/52) | - | - | - | - | 7 | - | - | - | - | - |  |
|  |  | 3IPV | tOPV | 5.8 | stool | culture | 3 | 10 (5/52) | - | - | - | - | 7 | - | - | - | - | - |  |
|  |  | 2IPV | tOPV | 6 | stool | culture | 1 | 18 (13/72) | - | - | - | - | 7 | - | - | - | - | - | 3.37 irrespective of serotype |
|  |  | 2IPV | tOPV | 5 | stool | culture | 2 | 93 (67/72) | - | - | - | - | 7 | - | - | - | - | - |  |
|  |  | 2IPV | tOPV | 5.8 | stool | culture | 3 | 14 (10/72) | - | - | - | - | 7 | - | - | - | - | - |  |
|  |  | unvaccinated | tOPV | 6 | stool | culture | 1 | 17 (9/54) | - | - | - | - | 7 | - | - | - | - | - | 3.89 irrespective of serotype |
|  |  | unvaccinated | tOPV | 5 | stool | culture | 2 | 89 (48/54) | - | - | - | - | 7 | - | - | - | - | - |  |
|  |  | unvaccinated | tOPV | 5.8 | stool | culture | 3 | 6 (3/54) | - | - | - | - | 7 | - | - | - | - | - |  |
| Ghendon et al 1961 [[9](#_ENREF_9)] | Russia | seronegative | mOPV1 | 6 | stool | culture | 1 | 80 (24/30) | - | - | - | - | - | - | - | - | - | 20 | 5.15 |
|  |  | 2IPV (seropositive) | mOPV1 | 6 | stool | culture | 1 | 68 (21/31) | - | - | - | - | - | - | - | - | - | 12 | 4.11 |
|  |  | 2OPV (seropositive) | mOPV1 | 6 | stool | culture | 1 | 36 (12/33) | - | - | - | - | - | - | - | - | - | 5 | 2.18 |
| Glezen et al. 1966 [[10](#_ENREF_10)] | USA | 0-6IPV^*^ | mOPV1 | 5.5 | stool | culture | 1 | 73 (133/181) | 17 (15/89) | 18 (17/92) | 10 (9/91) | - | 7 | 14 | 21 | 28 | - | 15 | 3 log10 pfu/g |
| Haas et al 1961 [[11](#_ENREF_11)] | Switzerland | unvaccinated | mOPV1 | 6.8 | stool | culture | 1 | 84 (36/43) | 75 (30/40) | 71 (32/45) | - | - | 5 | 10 | 25 | - | - | - | - |
|  |  | unvaccinated | mOPV2 | 6.8 | stool | culture | 2 | 76 (31/41) | 81 (34/42) | 95 (36/38) | - | - | 5 | 10 | 25 | - | - | - | - |
|  |  | unvaccinated | mOPV3 | 6.8 | stool | culture | 3 | 85 (33/39) | 89 (33/37) | 85 (34/40) | - | - | 5 | 10 | 25 | - | - | - | - |
| Henry et al 1966 [[12](#_ENREF_12)] | UK | unvaccinated | mOPV1 | 1.7-5.7 | stool | culture | 1 | 83 (40/48) | - | - | - | - | - | - | - | - | - | >18 days | median 4.1-5.0 |
|  |  | 3IPV | mOPV1 | 1.7-5.7 | stool | culture | 1 | 86 (42/49) | - | - | - | - | - | - | - | - | - | >18 days | median 4.1-5.0 |
|  |  | 4IPV | mOPV1 | 1.7-5.7 | stool | culture | 1 | 65 (28/43) | - | - | - | - | - | - | - | - | - | >18 days | median 4.1-5.0 |
|  |  | 3tOPV | mOPV1 | 1.7-5.7 | stool | culture | 1 | 32 (16/50) | - | - | - | - | - | - | - | - | - | 10-18 days | median <3.0 |
| John et al 1975 [[13](#_ENREF_13)] | India | seronegative | tOPV | 6 | stool | culture | 1 | 15 (10/66) | - | - | - | - | 7-14 | - | - | - | - | - | - |
|  |  | seronegative | tOPV | 5 | stool | culture | 2 | 48 (43/90) | - | - | - | - | 7-14 | - | - | - | - | - | - |
|  |  | seronegative | tOPV | 5.5 | stool | culture | 3 | 34 (31/90) | - | - | - | - | 7-14 | - | - | - | - | - | - |
| Kok et al 1982 [[14](#_ENREF_14)] | Czechoslovakia | 3tOPV | mOPV1 | 3.7 | stool | culture | 1 | 3 (2/60) | - | - | - | - | 7 | - | - | - | - | - | - |
|  |  | 3IPV | mOPV1 | 3.7 | stool | culture | 1 | 7 (6/84) | - | - | - | - | 7 | - | - | - | - | - | - |
| Kucharska et al 1985 [[15](#_ENREF_15)] | Czechoslovakia | unvaccinated | mOPV1 | 5 | stool | culture | 1 | 44 (16/36) | 33 (12/36) | 28 (10/36) | 28 (10/36) | - | 7 | 14 | 21 | 28 | - | 39 | 6.6 |
|  |  | unvaccinated | bOPV2,3 | 5 | stool | culture | 2 | 83 (30/36) | 64 (23/36) | 36 (13/36) | 33 (12/36) | - | 7 | 14 | 21 | 28 | - | 26 | 6.5 |
|  |  | unvaccinated | bOPV2,3 | 5.3 | stool | culture | 3 | 19 (7/36) | 28 (10/36) | 42 (15/36) | 36 (13/36) | - | 7 | 14 | 21 | 28 | - | - | 5.5 |
|  |  | 1mOPV1/1bOPV2,3 | mOPV1 | 5 | stool | culture | 1 | 17 (1/6) | 17 (1/6) | 33 (2/6) | 0 (0/6) | - | 7 | 14 | 21 | 28 | - | - | - |
|  |  | 1mOPV1/1bOPV2,3 | bOPV2,3 | 5 | stool | culture | 2 | 33 (2/6) | 0 (0/6) | 50 (3/6) | 17 (1/6) | - | 7 | 14 | 21 | 28 | - | - | - |
|  |  | 1mOPV1/1bOPV2,3 | bOPV2,3 | 5.3 | stool | culture | 3 | 33 (2/6) | 0 (0/6) | 50 (3/6) | 17 (1/6) | - | 7 | 14 | 21 | 28 | - | - | - |
| Laasri et al. 2005 [[16](#_ENREF_16)] | USA | unvaccinated | tOPV | 5.4 | stool | PCR | 1 | 42 (20/48) | 40 (19/48) | - | - | - | 7 | 21 | - | - | - | - | 627 (95% CI 398-987) geometric mean copy number irrespective of serotype |
|  |  | unvaccinated | tOPV | 4.5 | stool | PCR | 2 | 88 (42/48) | 58 (28/48) | - | - | - | 7 | 21 | - | - | - | 41 |  |
|  |  | unvaccinated | tOPV | 5.2 | stool | PCR | 3 | 58 (28/48) | 48 (23/48) | - | - | - | 7 | 21 | - | - | - | 71 |  |
|  |  | 2tOPV | tOPV | 5.4 | stool | PCR | 1 | 7 (3/41) | 2 (1/42) | - | - | - | 7 | 21 | - | - | - | 14 | - |
|  |  | 2tOPV | tOPV | 4.5 | stool | PCR | 2 | 10 (4/41) | 0 (0/42) | - | - | - | 7 | 21 | - | - | - | 4 | - |
|  |  | 2tOPV | tOPV | 5.2 | stool | PCR | 3 | 17 (7/41) | 5 (2/42) | - | - | - | 7 | 21 | - | - | - | 11 | - |
|  |  | 2IPV | tOPV | 5.4 | stool | PCR | 1 | 55 (23/42) | 26 (10/38) | - | - | - | 7 | 21 | - | - | - | 19 | 155 (95% CI 53-456) geometric mean copy number irrespective of serotype |
|  |  | 2IPV | tOPV | 4.5 | stool | PCR | 2 | 76 (32/42) | 26 (10/38) | - | - | - | 7 | 21 | - | - | - | 19 |  |
|  |  | 2IPV | tOPV | 5.2 | stool | PCR | 3 | 50 (21/42) | 21 (8/38) | - | - | - | 7 | 21 | - | - | - | 16 |  |
| Maldonado et al. 1997 [[17](#_ENREF_17)] | Mexico | unvaccinated | tOPV | 6 | stool | culture | 1 | 53 (96/181) | 33 (59/181) | - | - | - | 7 | 21 | - | - | - | 29 | - |
|  |  | unvaccinated | tOPV | 5 | stool | culture | 2 | 45 (81/181) | 34 (62/181) | - | - | - | 7 | 21 | - | - | - | 52 | - |
|  |  | unvaccinated | tOPV | 5.6 | stool | culture | 3 | 12 (21/181) | 18 (33/181) | - | - | - | 7 | 21 | - | - | - | - | - |
|  |  | 1tOPV | tOPV | 6 | stool | culture | 1 | 10 (18/181) | 4 (7/181) | - | - | - | 7 | 21 | - | - | - | 14 | - |
|  |  | 1tOPV | tOPV | 5 | stool | culture | 2 | 1 (2/181) | 3 (5/181) | - | - | - | 7 | 21 | - | - | - | 14 | - |
|  |  | 1tOPV | tOPV | 5.6 | stool | culture | 3 | 22 (40/181) | 5 (9/181) | - | - | - | 7 | 21 | - | - | - | 9 | - |
| Modlin et al 1997 [[18](#_ENREF_18)] | USA | 3IPV | tOPV | 6.5 | stool | culture | 1 | 18 (13/74) | - | - | - | - | 3-21 | - | - | - | - | - | - |
|  |  | 3IPV | tOPV | 5.4 | stool | culture | 2 | 39 (29/74) | - | - | - | - | 3-21 | - | - | - | - | - | - |
|  |  | 3IPV | tOPV | 6.3 | stool | culture | 3 | 78 (58/74) | - | - | - | - | 3-21 | - | - | - | - | - | - |
|  |  | 3tOPV | tOPV | 6.5 | stool | culture | 1 | 4 (3/73) | - | - | - | - | - | - | - | - | - | - | - |
|  |  | 3tOPV | tOPV | 5.4 | stool | culture | 2 | 3 (2/73) | - | - | - | - | - | - | - | - | - | - | - |
|  |  | 3tOPV | tOPV | 6.3 | stool | culture | 3 | 10 (7/73) | - | - | - | - | - | - | - | - | - | - | - |
|  |  | 2IPV/1tOPV | tOPV | 6.5 | stool | culture | 1 | 27 (21/79) | - | - | - | - | - | - | - | - | - | - | - |
|  |  | 2IPV/1tOPV | tOPV | 5.4 | stool | culture | 2 | 11 (9/79) | - | - | - | - | - | - | - | - | - | - | - |
|  |  | 2IPV/1tOPV | tOPV | 6.3 | stool | culture | 3 | 54 (43/79) | - | - | - | - | - | - | - | - | - | - | - |
|  |  | 2IPV/2tOPV | tOPV | 6.5 | stool | culture | 1 | 14 (11/80) | - | - | - | - | - | - | - | - | - | - | - |
|  |  | 2IPV/2tOPV | tOPV | 5.4 | stool | culture | 2 | 4 (3/80) | - | - | - | - | - | - | - | - | - | - | - |
|  |  | 2IPV/2tOPV | tOPV | 6.3 | stool | culture | 3 | 20 (16/80) | - | - | - | - | - | - | - | - | - | - | - |
|  |  | 2IPV/3tOPV | tOPV | 6.5 | stool | culture | 1 | 14 (10/70) | - | - | - | - | - | - | - | - | - | - | - |
|  |  | 2IPV/3tOPV | tOPV | 5.4 | stool | culture | 2 | 3 (2/70) | - | - | - | - | - | - | - | - | - | - | - |
|  |  | 2IPV/3tOPV | tOPV | 6.3 | stool | culture | 3 | 17 (12/70) | - | - | - | - | - | - | - | - | - | - | - |
| Mohammed et al 2010 [[19](#_ENREF_19)] | Oman | 3IPV | mOPV1 | 6 | stool | culture | 1 | 63 (99/157) | - | - | - | - | 7 | - | - | - | - | - | - |
| Onorato et al 1991 [[20](#_ENREF_20)] | USA | 3tOPV | mOPV1 | 2.7-5.7 | stool | culture | 1 | 11 (9/79) | 3 (2/79) | 1 (1/79) | - | - | 7 | 14 | 21 | - | - | 6 | 2.54 log10 pfu/g |
|  |  | 3IPV | mOPV1 | 2.7-5.7 | stool | culture | 1 | 52 (48/93) | 32 (30/93) | 12 (11/93) | - | - | 7 | 14 | 21 | - | - | 16 | 3.24 log10 pfu/g |
| Piirainen et al 1998 [[21](#_ENREF_21)]  Public Health Laboratory Service (PHLS) 1965 [[22](#_ENREF_22)] | Finland | 3IPV | mOPV3 | 5.7 | stool | culture | 3 | 93 (41/44) | 87 (39/45) | 82 (36/44) | 73 (32/44) | 55 (24/44) | 8 | 12 | 16 | 21 | 28 | 62 | 5.5 TCID50 /cm3 faeces |
|  | UK | unvaccinated | mOPV1 | 4.7 | stool | culture | 1 | 83 (19/23) | - | - | - | - | - | - | - | - | - | - | - |
|  |  | 3IPV | mOPV1 | 4.7 | stool | culture | 1 | 80 (55/69) | - | - | - | - | - | - | - | - | - | - | - |
|  |  | 3tOPV | mOPV1 | 4.7 | stool | culture | 1 | 34 (18/53) | - | - | - | - | - | - | - | - | - | - | - |
| Roca-Garcia et al 1964 [[23](#_ENREF_23)] | Costa Rica | seronegative | tOPV | - | swab | culture | 1 | 58 (61/106) | - | - | - | - | 14-21 | - | - | - | - | - | - |
|  |  | seronegative | tOPV | - | swab | culture | 2 | 1 (1/67) | - | - | - | - | 14-21 | - | - | - | - | - | - |
|  |  | seronegative | tOPV | - | swab | culture | 3 | 17 (5/29) | - | - | - | - | 14-21 | - | - | - | - | - | - |
| Sutter et al 2010 [[24](#_ENREF_24)] | India | 1mOPV1 | mOPV1 | 6 | stool | culture | 1 | 66 (111/168) | - | - | - | - | 7 | - | - | - | - | - | - |
|  |  | 1mOPV2 | mOPV2 | 5 | stool | culture | 2 | 74 (126/170) | - | - | - | - | 7 | - | - | - | - | - | - |
|  |  | 1mOPV3 | mOPV3 | 5.8 | stool | culture | 3 | 73 (120/165) | - | - | - | - | 7 | - | - | - | - | - | - |
|  |  | 1tOPV | tOPV | 6 | stool | culture | 1 | 49 (83/168) | - | - | - | - | 7 | - | - | - | - | - | - |
|  |  | 1tOPV | tOPV | 5 | stool | culture | 2 | 68 (114/168) | - | - | - | - | 7 | - | - | - | - | - | - |
|  |  | 1tOPV | tOPV | 5.8 | stool | culture | 3 | 26 (44/168) | - | - | - | - | 7 | - | - | - | - | - | - |
|  |  | 1bOPV1,3 | bOPV1,3 | 6 | stool | culture | 1 | 65 (103/159) | - | - | - | - | 7 | - | - | - | - | - | - |
|  |  | 1bOPV1,3 | bOPV1,3 | 5.8 | stool | culture | 3 | 50 (80/159) | - | - | - | - | 7 | - | - | - | - | - | - |
| Sutter et al 2000 [[25](#_ENREF_25)] | Oman | 5tOPV/1IPV | mOPV3 | 6.12 | stool | culture | 3 | 13 (20/157) | - | - | - | - | 7 | - | - | - | - | - | - |
|  |  | 6tOPV | mOPV3 | 6.12 | stool | culture | 3 | 17 (46/275) | - | - | - | - | 7 | - | - | - | - | - | - |
|  |  | 5tOPV/1mOPV3 | mOPV3 | 6.12 | stool | culture | 3 | 9 (15/157) | - | - | - | - | 7 | - | - | - | - | - | - |
| Swartz et al 2008 [[26](#_ENREF_26)] | Israel | 3tOPV/3IPV | tOPV | 6 | stool | culture | 1 | 36 (27/75) | 12 (6/51) | 15 (7/47) | 10 (4/42) | - | 7 | 14 | 21 | 28 | - | 11 | 3-7 at 7 days and 2-5 at 21 days irrespective of serotype or vaccine schedule |
|  |  | 3tOPV/3IPV | tOPV | 5 | stool | culture | 2 | 19 (14/75) | 6 (3/51) | 0 (0/47) | 0 (0/42) | - | 7 | 14 | 21 | 28 | - | 5 |  |
|  |  | 3tOPV/3IPV | tOPV | 5.8 | stool | culture | 3 | 25 (19/75) | 22 (11/51) | 9 (4/47) | 5 (2/42) | - | 7 | 14 | 21 | 28 | - | 17 |  |
|  |  | 2tOPV/2IPV | tOPV | 6 | stool | culture | 1 | 35 (29/82) | 27 (13/49) | 14 (6/44) | 3 (1/40) | - | 7 | 14 | 21 | 28 | - | 14 |  |
|  |  | 2tOPV/2IPV | tOPV | 5 | stool | culture | 2 | 24 (20/82) | 4 (2/49) | 5 (2/44) | 0 (0/40) | - | 7 | 14 | 21 | 28 | - | 5 |  |
|  |  | 2tOPV/2IPV | tOPV | 5.8 | stool | culture | 3 | 27 (22/82) | 27 (13/49) | 14 (6/44) | 3 (1/40) | - | 7 | 14 | 21 | 28 | - | 20 |  |
| Swartz et al 1972 [[27](#_ENREF_27)] | Israel | unvaccinated | tOPV | 6 | stool | culture | 1 | 46 (104/226) | - | - | - | - | 7 | - | - | - | - | - | - |
|  |  | unvaccinated | tOPV | 5 | stool | culture | 2 | 77 (175/226) | - | - | - | - | 7 | - | - | - | - | - | - |
|  |  | unvaccinated | tOPV | 5.5 | stool | culture | 3 | 42 (94/226) | - | - | - | - | 7 | - | - | - | - | - | - |
|  |  | 2tOPV | tOPV | 6 | stool | culture | 1 | 12 (26/226) | - | - | - | - | 7 | - | - | - | - | - | - |
|  |  | 2tOPV | tOPV | 5 | stool | culture | 2 | 10 (22/226) | - | - | - | - | 7 | - | - | - | - | - | - |
|  |  | 2tOPV | tOPV | 5.5 | stool | culture | 3 | 14 (31/226) | - | - | - | - | 7 | - | - | - | - | - | - |
| Urosawa et al 1963 [[28](#_ENREF_28)] | Japan | unvaccinated | mOPV1 | 7.4 | stool | culture | 1 | 23 (28/124) | - | - | - | - | 28 | - | - | - | - | - | - |
|  |  | unvaccinated | mOPV2 | 7.6 | stool | culture | 2 | 11 (13/119) | - | - | - | - | 28 | - | - | - | - | - | - |
|  |  | unvaccinated | mOPV3 | 7.9 | stool | culture | 3 | 20 (23/115) | - | - | - | - | 28 | - | - | - | - | - | - |
| WHO collaborative group 1997a [[29](#_ENREF_29)] | Gambia | 3IPV | mOPV1 | 6 | stool | culture | 1 | 16 (18/112) | - | - | - | - | 7 | - | - | - | - | - | - |
|  |  | 4tOPV/3IPV | mOPV1 | 6 | stool | culture | 1 | 9 (10/111) | - | - | - | - | 7 | - | - | - | - | - | - |
|  |  | 4tOPV | mOPV1 | 6 | stool | culture | 1 | 4 (4/111) | - | - | - | - | 7 | - | - | - | - | - | - |
| WHO collaborative group 1997b [[29](#_ENREF_29)] | Oman | 3IPV | mOPV1 | 6 | stool | culture | 1 | 10 (18/177) | - | - | - | - | 7 | - | - | - | - | - | - |
|  |  | 4tOPV/3IPV | mOPV1 | 6 | stool | culture | 1 | 11 (19/177) | - | - | - | - | 7 | - | - | - | - | - | - |
|  |  | 4tOPV | mOPV1 | 6 | stool | culture | 1 | 13 (23/177) | - | - | - | - | 7 | - | - | - | - | - | - |
| WHO collaborative group 1997c [[29](#_ENREF_29)] | Thailand | 3IPV | mOPV1 | 6 | stool | culture | 1 | 57 (75/132) | - | - | - | - | 7 | - | - | - | - | - | - |
|  |  | 4tOPV/3IPV | mOPV1 | 6 | stool | culture | 1 | 14 (19/133) | - | - | - | - | 7 | - | - | - | - | - | - |
|  |  | 4tOPV | mOPV1 | 6 | stool | culture | 1 | 14 (19/133) | - | - | - | - | 7 | - | - | - | - | - | - |

^*^children in these 2 studies had received different numbers of IPV doses within the reported range but shedding data were not disaggregated by number of doses

**References**

1. Asturias EJ, Dueger EL, Omer SB, et al. Randomized trial of inactivated and live polio vaccine schedules in Guatemalan infants. J Infect Dis **2007**; 196:692-8.

2. Bauer P. [Excretion values following Sabin oral vaccination against poliomyelitis in Tyrol]. Archiv Fur Hygiene Und Bakteriologie **1968**; 152:410-4.

3. Biffi MR, Tagger A, Bruscolini F, Salvaggio L, Albano A. [Fecal excretion of poliovirus in subjects subjected to antipoliomyelitis vaccination]. Nuovi Ann Ig Microbiol **1982**; 33:285-94.

4. Dong DX, Hu XM, Liu WJ, et al. Immunization of neonates with trivalent oral poliomyelitis vaccine (Sabin). Bull World Health Organ **1986**; 64:853-60.

5. du Chatelet IP, Merchant AT, Fisher-Hoch S, et al. Serological response and poliovirus excretion following different combined oral and inactivated poliovirus vaccines immunization schedules. Vaccine **2003**; 21:1710-8.

6. El-Sayed N, El-Gamal Y, Abbassy AA, et al. Double-blind randomized controlled clinical trial of monovalent type 1 oral poliovirus vaccine. N Engl J Med **2008**; 359:1655-65.

7. Enders-Ruckle G, Siegert R. [Viral excretion and antibody formation after use of a live trivalent poliomyelitis vaccine (Cox-Lederle)]. Dtsch Med Wochenschr **1961**; 86:1999-2008.

8. Galindo M, Lago PM, Caceres V, Landaverde M, Sutter R. (Cuba IPV Study Collaborative Group). Randomized, placebo-controlled trial of inactivated poliovirus vaccine in Cuba. New Engl J Med **2007**; 356:1536-44.

9. Ghendon YZ, Sanakoyeva II. Comparison of the resistance of the intestinal tract to poliomyelitis virus (Sabin’s strains) in persons after naturally and experimentally acquired immunity. Acta Virol **1961**; 5:265-73.

10. Glezen WP, Lamb GA, Belden EA, Chin TDY. Quantitiative relationship of preexisting homotypic antibodies to the excretion of attenuated poliovirus type 1. Am J Epidemiol **1966**; 83:224-37.

11. Haas R, Dostal V, Lindenmann J, Maass G, Thomssen R. [Virological research after oral poliomyelitis vaccination (Sabin). 1. Research on viral excretion, antibody formation and frequency of contact infections]. Dtsch Med Wochenschr **1961**; 86:2413-21.

12. Henry JL, Jaikaran ES, Davies JR, et al. A study of poliovaccination in infancy: excretion following challenge with live virus by children given killed or living poliovaccine. J Hyg (Lond) **1966**; 64:105-20.

13. John TJ. Oral polio vaccination of children in the tropics. II. Antibody response in relation to vaccine virus infection. Am J Epidemiol **1975**; 102:414-21.

14. Kok PW, Leeuwenburg J, Tukei P, et al. Serological and virological assessment of oral and inactivated poliovirus vaccines in a rural population in Kenya. Bull WHO **1992**; 70:93-103.

15. Kucharska Z. Excretion of live attenuated polioviruses in the faeces of orally vaccinated children. Comparison of two immunization schedules. J Hyg Epidemiol Microbiol Immunol **1985**; 29:211-8.

16. Laassri M, Lottenbach K, Belshe R, et al. Effect of different vaccination schedules on excretion of oral poliovirus vaccine strains. J Infect Dis **2005**; 192:2092-8.

17. Maldonado YA, PenaCruz V, Sanchez MD, et al. Host and viral factors affecting the decreased immunogenicity of Sabin type 3 vaccine after administration of trivalent oral polio vaccine to rural Mayan children. J Infect Dis **1997**; 175:545-53.

18. Modlin JF, Halsey NA, Thoms ML, Meschievitz CK, Patriarca PA. Humoral and mucosal immunity in infants induced by three sequential inactivated poliovirus vaccine - Live attenuated oral poliovirus vaccine immunization schedules. J Infect Dis **1997**; 175:S228-S34.

19. Mohammed AJ, AlAwaidy S, Bawikar S, et al. Fractional doses of inactivated poliovirus vaccine in Oman. N Engl J Med **2010**; 362:2351-9.

20. Onorato IM, Modlin JF, McBean AM, Thoms ML, Losonsky GA, Bernier RH. Mucosal immunity induced by enhanced-potency inactivated and oral polio vaccines. J Infect Dis **1991**; 163:1-6.

21. Piirainen L, Stenvik M, Roivainen M, Eskola J, Beuvery EC, Hovi T. Randomised, controlled trial with the trypsin-modified inactivated poliovirus vaccine: assessment of intestinal immunity with live challenge virus. Vaccine **1999**; 17:1084-90.

22. Public Health Laboratory Service. The excretion of type 1 poliovirus after challenge following primary immunization with quadruple, Salk and Sabin vaccines. Mon Bull Minist Health Public Health Lab Serv **1965**; 24:365-9.

23. Roca-Garcia M, Markham FS, Cox HR, Vargas-Mendez O, Guevara EC, Montoya JA. Poliovirus shedding and seroconversion. Studies of 816 Costa Rican children fed trivalent vaccine. JAMA **1964**; 188:639-46.

24. Sutter RW, John TJ, Jain H, et al. Immunogenicity of bivalent types 1 and 3 oral poliovirus vaccine: a randomised, double-blind, controlled trial. The Lancet **2010**; 376:1682-8.

25. Sutter RW, Suleiman AJM, Malankar P, et al. Trial of a supplemental dose of four poliovirus vaccines. New Engl J Med **2000**; 343:767-73.

26. Swartz TA, Green MS, Handscher R, et al. Intestinal immunity following a combined enhanced inactivated polio vaccine/oral polio vaccine programme in Israel. Vaccine **2008**; 26:1083-90.

27. Swartz TA, Skalska P, Gerichte.Cg, Cockburn WC. Routine administration of oral polio vaccine in a subtropical area. Factors possibly influencing seroconversion rates. J Hyg (Lond) **1972**; 70:719-26.

28. Urasawa S, Ogawa M, Kanno T. Field trial with live oral poliovirus vaccine. I. Schedule of this trial and excretion of virus in feces after the administration of monovalent oral poliovirus vaccine. Sapporo Igaku Zasshi **1963**; 23:194-201.

29. World Health Organization Collaborative Study Group on Oral Poliovirus Vaccine. Factors affecting the immunogenicity of oral poliovirus vaccine - a prospective evaluation in Brazil and the Gambia. J Infect Dis **1995**; 171:1097-106.
