# Supplementary material for: Systematic Review of Mucosal Immunity Induced by Oral and Inactivated Poliovirus Vaccines against Virus Shedding following Oral Poliovirus Challenge
Source: PLoS Pathog. 2012 Apr 19;8(4):e1002599. doi: 10.1371/journal.ppat.1002599 (PMC3330118; doi:10.1371/journal.ppat.1002599)
Supplement: Table S2 — Studies included in the systematic review that examined poliovirus shedding in nasopharyngeal secretions after administration of OPV. Vaccination schedules are given as the number of doses followed by the type of vaccine. tOPV = trivalent OPV, mOPV1 = serotype 1 monovalent OPV. (DOCX) [file ppat.1002599.s003.docx]

**Table S2** Studies included in the systematic review that examined poliovirus shedding in nasopharyngeal secretions after administration of OPV. Vaccination schedules are given as the number of doses followed by the type of vaccine. tOPV = trivalent OPV, mOPV1 = serotype 1 monovalent OPV.

| **Study** | **location** | **schedule** | **challenge vaccine** | **minimum titer of challenge poliovirus (log10 TCID50)** | **sample** | **detection** | **serotype** | **Proportion shedding according % (number)** | **Time of sample (days after challenge)** |
| --- | --- | --- | --- | --- | --- | --- | --- | --- | --- |
| Bauer et al 1968 [[1](#_ENREF_1)] | Austria | 0-3IPV^*^ | tOPV | NA | washing | culture | 1 | 9 (6/65) | 7 |
|  |  | 0-3IPV^*^ | tOPV | NA | washing | culture | 2 | 2 (1/50) | 7 |
|  |  | 0-3IPV^*^ | tOPV | NA | washing | culture | 3 | 2 (1/56) | 7 |
| Glezen et al 1966 [[2](#_ENREF_2)] | USA | 0-6IPV^*^ | mOPV1 | 5.5 | washing | culture | 1 | 10 (9/92) | 3-7 |
| Kok et al 1992 [[3](#_ENREF_3)] | Kenya | 3tOPV | mOPV1 | 3.5 | washing | culture | 1 | 0 (0/60) | 7 |
|  |  | 2-3IPV | mOPV1 | 3.5 | washing | culture | 1 | 0 (0/84) | 7 |
| Onorato et al 1991 [[4](#_ENREF_4)] | USA | 3tOPV | mOPV1 | 2.7-5.7 | swab | culture | 1 | 3 (2/78) | 7 |
|  |  | 3IPV | mOPV1 | 2.7-5.7 | swab | culture | 1 | 1 (1/91) | 7 |

^*^children in these 2 studies had received different numbers of IPV doses within the reported range but shedding data were not disaggregated by number of doses

**References**

1. Bauer P. [Excretion values following Sabin oral vaccination against poliomyelitis in Tyrol]. Archiv Fur Hygiene Und Bakteriologie **1968**; 152:410-4.

2. Glezen WP, Lamb GA, Belden EA, Chin TDY. Quantitiative relationship of preexisting homotypic antibodies to the excretion of attenuated poliovirus type 1. Am J Epidemiol **1966**; 83:224-37.

3. Kok PW, Leeuwenburg J, Tukei P, et al. Serological and virological assessment of oral and inactivated poliovirus vaccines in a rural population in Kenya. Bull WHO **1992**; 70:93-103.

4. Onorato IM, Modlin JF, McBean AM, Thoms ML, Losonsky GA, Bernier RH. Mucosal immunity induced by enhanced-potency inactivated and oral polio vaccines. J Infect Dis **1991**; 163:1-6.
